# Service users' experiences of, and engagement with, a nationally implemented digital diabetes prevention programme

**DOI:** 10.1111/bjhp.12787

**Published:** 2025-02-19

**Authors:** Davide Moussas‐Alvarez, Rhiannon E. Hawkes, Lisa M. Miles, Charlotte Dack, David P. French

**Affiliations:** ^1^ Department of Psychology, Centre for Motivation and Health Behaviour Change University of Bath Bath UK; ^2^ Division of Psychology and Mental Health, School of Health Sciences, Faculty of Biology, Medicine and Health, Manchester Centre for Health Psychology University of Manchester Manchester UK

**Keywords:** behaviour change, diabetes prevention, digital interventions, qualitative, thematic analysis, user engagement, user experiences

## Abstract

**Objectives:**

Type 2 diabetes mellitus (T2DM) may be prevented by promoting weight loss through adopting healthier behaviours (e.g., improved diet and increased physical activity). In 2016, the National Health Service (NHS) in England introduced a 9‐month face‐to‐face T2DM prevention intervention, delivered by four independent providers. Since 2019, the NHS Digital Diabetes Prevention Programme (NHS‐DDPP) was offered to increase accessibility of the programme. This research aimed to understand how service users engaged with, and experienced using the NHS‐DDPP.

**Design:**

Qualitative interviews.

**Method:**

Semi‐structured interviews were conducted with service users (*n* = 45) who took part in one of the four NHS‐DDPP providers' programmes and transcribed verbatim. Transcripts were thematically analysed using a framework approach.

**Results:**

Two overarching themes were produced: ‘Personalized Guidance’ and ‘Path to Success’. Service users valued having health coach support, which provided personalized guidance throughout the programme, alongside access to different app features to suit their needs (e.g., educational content, tracking health behaviours, group support). Service users described self‐monitoring, feedback from their health coach and support from their social circle as helpful towards changing their health behaviours. This enabled them to visualize their progress and provided accountability.

**Conclusion:**

Service users emphasized how human contact alongside this digital behaviour change programme improved their experiences and engagement with the programme. Digital health interventions could consider how to better incorporate support from health coaches, friends and family to help users in making behavioural changes. Future digital health interventions should consider how best to harness non‐digital elements to promote behaviour change.

## INTRODUCTION

People with type 2 diabetes mellitus (T2DM) have increased levels of glucose in their blood which can cause complications, such as heart disease, stroke, and kidney failure (Balaji et al., 2019). The number of deaths caused by T2DM worldwide has doubled since the year 2000, putting T2DM in the top 10 leading causes of death in the world (WHO, 2020). The prevalence of T2DM is at a new high in the United Kingdom (UK) with more than 5 million people being diagnosed or at risk of developing T2DM (Diabetes UK, 2023). Given the prevalence of T2DM and the associated complications, its prevention is a public health priority. Around the world, several national diabetes prevention programmes have been implemented (e.g., Ramachandran et al., 2006; Saaristo et al., 2010; Yin et al., 2016). These programmes promote weight loss and reduce blood glucose levels through behavioural changes such as improved diet and increased physical activity to delay or prevent the onset of T2DM (Ashra et al., 2015).

In England, the ‘Healthier You: National Health Service Diabetes Prevention Programme’ (NHS‐DPP) was rolled out in 2016 for adults at risk of developing T2DM (NHS England, 2017). The programme has been implemented nationally and delivered by multiple independent service providers, who each offer a 9‐month face‐to‐face intervention targeting physical activity and food choices to promote weight loss. The NHS‐DPP is underpinned by a service specification (NHS England, 2019) that specified behaviour change techniques (BCTs) that providers should include in their interventions (e.g., goal setting, action planning, self‐monitoring), based on the current available evidence base (NICE, 2017). Evaluations of the NHS‐DPP have found the programme to be effective in promoting weight loss and lowering blood glucose levels in those who attended the programme (Marsden et al., 2022; Ravindrarajah et al., 2023; Valabhji et al., 2020).

However, a large proportion of people who were referred to the programme did not take it up (Valabhji et al., 2020). One reason might be that face‐to‐face interventions are not suitable for everyone (e.g., working adults, people living with disabilities, or people living in remote locations), due to many different barriers including perceived or physical capabilities (e.g., lack of time or transport) (Ritchie et al., 2021; Shawley‐Brzoska & Misra, 2018). Digital services could therefore be beneficial for those who cannot or do not want to attend face‐to‐face programmes (McGough et al., 2019). Digital programmes come with their own accessibility issues, for example, people with a low health literacy or a lower socioeconomic status background may not have access to the adequate technology (Estacio et al., 2019). Given this, implementing both face‐to‐face and digital programmes can accommodate a wider number of people, and tackle different sources of inequality (Ross et al., 2022).

In 2017 NHS England rolled out a pilot programme of a digital version of the NHS‐DPP (Murray et al., 2019), and evaluations of this found that users of the programme had a clinically significant reduction in weight and HbA1c over 12 months (Ross et al., 2022). Consequently, in 2019, the NHS Digital Diabetes Prevention Programme (NHS‐DDPP) was launched nationally, whereby four independent digital providers were commissioned to deliver the intervention (NHS England, 2019). Providers delivered this programme via mobile and web applications which included educational content (e.g., articles and videos), health coach support (e.g., via calls or messaging) and group support (access to chats or forums with other users) (Hawkes, Miles, & French, 2023). The programme was underpinned by the same Service Specification as the face‐to‐face NHS‐DPP, meaning the same BCTs were specified to be included in the NHS‐DDPP. An assessment of the fidelity of the behaviour change content found that fidelity was higher in the NHS‐DDPP (Hawkes et al., 2022) compared with the face‐to‐face service (Hawkes et al., 2020). Consequently, if service users fully engage with the digital version, the programme should achieve higher exposure to behaviour change content (Hawkes et al., 2022).

However, the NHS Service Specification (NHS England, 2017) provided a flexible framework which allowed the four digital providers to deliver their own versions of the NHS‐DDPP (Miles et al., 2023a), described in Table 1. These differences in digital programme features could influence the effectiveness of the programme and issues could arise in the delivery of these digital features in the real world (Fleming et al., 2018; Hawkes, Miles, & French, 2023).

**TABLE 1:** Modes of delivery of the NHS‐DDPP by provider.

Digital programme feature	NHS‐DDPP provider programme			
	Provider 1	Provider 2	Provider 3	Provider 4
App functions	Tracking behaviours and outcomes Setting goals Feedback on behaviours and outcomes via graphs Leader board	Tracking behaviours and outcomes Setting goals and tracking progress against goals Feedback on behaviours and outcomes via graphs	Tracking behaviours and outcomes Setting goals Feedback on behaviours and outcomes via graphs and health coach	Tracking behaviours and outcomes Barcode scanner Feedback on behaviours and outcomes via graphs
Educational content	Articles, videos and podcasts unlocked (first 3 months) on app	PDFs, videos, links sent from health coach via messaging on app	Online learning platform where articles are unlocked over time on app	PDF workbook and weekly article on app
Group support	Group chats with 10–15 people and open group discussion forums	Forums for NHS‐DDPP members	No^a^	Open group discussion forums (akin to social media)
Health coach support	Messaging only (proactive health coach, weekly for 3 months)	Telephone/video calls and messaging (weekly for 4 weeks, bi‐weekly for 4 weeks, then monthly for 4 weeks)	Telephone calls and messaging (weekly, gradually reduced to monthly)	Messaging only (reactive health coach)

*Note*: Providers are labelled 1, 2, 3 and 4 in this manuscript to preserve anonymity for provider organizations. The labels 1, 2, 3 and 4 do not correspond to labels A, B, C, D used in a previously published article (Miles et al., 2023a).

^a^ At the time of data collection, no group support was delivered by Provider 3. This provider has since introduced a ‘group pathway’ that includes a group support function.

Understanding service users' experiences of the different digital features of NHS‐DDPP could provide new insight into reasons for a programme's effectiveness (Moore et al., 2015) and inform future developments of the programme, including determining reasons for high or low engagement with certain programme features. To date some research has explored user experiences with digital T2DM self‐management programmes. For example, users of a T2DM self‐management app expressed utilizing different intervention components that aligned with their self‐management needs (Baptista et al., 2020). Another recent interview study explored user experiences of a nationally implemented T2DM digital self‐management intervention in England; users reported disengaging with the structured education curriculum when they encountered education content that was not applicable to them and reported wanting more tailored feedback on the behaviours and outcomes they were monitoring in the programme (Hawkes et al., 2024). Both of these studies highlight the potential of harnessing the personalisation that digital programmes can offer to help people manage their health behaviours.

Some research has explored service users' engagement with the NHS‐DDPP. An analysis of usage data for the 9‐month programme found that self‐monitoring of behaviours (e.g., diet, physical activity) via the apps were regularly used, though other programme features such as group support ware rarely engaged with (Hawkes, Miles, Ainsworth, et al., 2023). A more recent qualitative study of the service users' experience with the online group support of the NHS‐DDPP identified that closed group chats received more user engagement compared with open group discussion forums (Cheung et al., 2024). Providers of the NHS‐DDPP have been continuously improving and developing their services based on users' experience feedback (Miles et al., 2023b). However, there is currently a dearth of investigation of service users' overall experiences of the digital programme. This study therefore aimed to understand how service users engaged with and experienced the NHS‐DDPP and to understand any variations across the provider programmes which may impact on engagement or experience. Specific research questions were:

1. How do service users describe their engagement with the NHS‐DDPP?
2. What are the service users' experiences with the NHS‐DDPP?
3. How do service users' engagement and experiences vary across providers?

## METHODS

Methods are reported in accordance with the Standards for Reporting Qualitative Research (SRQR) checklist (O'Brien et al., 2014). See File S1.

### Study design

This research employed a qualitative research design, where semi‐structured interviews asked service users about their experiences of the NHS‐DDPP when they were 2–4 months into the 9‐month programme.

### Participants

Participants were over the age of 18 years and had a pre‐diabetes diagnosis (HbA1c of 42–47 mmol/mol) in the last 24 months leading them to take up the NHS‐DDPP. Participants were only eligible to take part in this research if they could speak English and were able to provide full verbal consent.

### Procedures

Participants were invited to take part between February and April 2021. The four providers sent out the invitations to potential participants, along with details about the study including a participant information sheet. Any interested service users contacted the research team to arrange an interview. During recruitment, researchers used a stratified purposive sampling approach whereby follow‐up recruitment emails from providers targeted specific characteristics to aim for a diverse range of participant recruitment across providers, age, sex, and ethnic group. Further details on sampling and recruitment are provided in File S2 and a recent study (Miles et al., 2023a).

Interviews were conducted via telephone by two members of the research team (REH and LMM) who were both female Research Associates with training and experience in conducting and reporting qualitative methods. Full audio‐recoded verbal consent was obtained via telephone prior to commencing the interviews and socio‐demographic characteristics were recorded (age, sex, ethnicity and postcode). Researchers described the aims of the research to participants in lay terms, which were to understand their experiences of and engagement with the NHS‐DDPP, and how their participation in the programme may have helped them to make changes to their health behaviours. The interviews were audio‐recoded using an encrypted audio‐recorder, which lasted between 30 and 60 minutes. Interviews were then transcribed verbatim using an externally approved transcription service and pseudonymized for analysis. Field notes were made following each interview and recruitment was stopped when the researchers (REH, LMM, DPF) felt that no new content was being discussed (in what became the final two interviews for each provider), according to the concept of ‘information power’ which determines the adequacy and richness of the data to comprehensively answer the research questions (Malterud et al., 2016).

### Topic guide

The topic guide was developed to ask participants how they understood and used specific BCTs in the programme (goal setting, action planning, self‐monitoring, problem solving, feedback), the results of which have been reported separately (Miles et al., 2023a). Questions also asked service users about their experiences of using different components of the digital programme and their engagement with these programme features, results of which are reported in this article. The researchers, with the help of a Public and Patient Involvement and Engagement (PPIE) group (*n* = 4 people at risk of or living with T2DM; *n* = 2 female and *n* = 2 male), developed these questions and amended the original topic guide. The group advised on the wording of the questions and any additional questions to ask. For example, the following question was added to the topic guide as a result of obtaining feedback from the PPIE group: ‘Before you started the “Healthier You” digital sessions, what did you expect from the online course?’ See File S3 for the topic guide and File S4 for further information on the involvement of the PPIE group in this study, structured using the GRIPP2 reporting checklist (Staniszewska et al., 2017).

### Data analysis

Data were analysed using the framework approach to thematic analysis (Gale et al., 2013), to allow researchers to understand participants' views and experiences of specific features of the digital programme. This involved the development of a framework matrix to compare findings across participants and provider programmes on key programme features where relevant. Data were analysed using realist approach (Wiltshire & Ronkainen, 2021), to directly reflect the truth of the participants' experiences (Wiltshire, 2018). During the analysis, data were read and analysed provider by provider, which allowed the research team to better understand the differences and similarities between provider programmes.

The lead author (DMA) read all the interview transcripts and annotated any potential codes, themes or initial ideas about the data. An audit trail was kept throughout the analysis. The coding framework was developed by authors DMA, REH and DPF, based on digital features of the NHS‐DDPP (e.g., educational content, group support, health coach support and app usage) that were identified in a previous analysis (Hawkes, Miles, Ainsworth, et al., 2023; Hawkes, Miles, & French, 2023). This informed the development of a priori codes. Discussions were held between all authors to achieve consensus on the final coding framework. Additional codes were also developed inductively during data analysis to capture nuances in the data. See File S5 for the coding framework.

The coding process was facilitated using NVivo (QSR International Ltd., 2020) and charted into the framework by one researcher (DMA), with regular analysis meetings with a second researcher (REH). After the data were coded, eight different framework matrices were exported from NVivo to Excel (one for engagement and one for experiences for each provider), which were then summarized in two final frameworks: one for engagement and one for experience. For the purpose of this framework analysis, ‘engagement’ was conceptualized as service users' description of the extent to which they used features of the app (e.g., frequency and duration of use of components) or how much they interacted with health coaches or other users on the programme. ‘Experience’ was conceptualized as service users' subjective experiences with the intervention components and the overall programme (e.g., positive and negative experiences with specific intervention components such as education content, tracking features or support). See also File S5. Data were interpreted using these final frameworks, where potential themes were finalized following discussion among all authors. File S6 includes a sample of the ‘engagement’ and ‘experience’ summary frameworks. Themes and sub‐themes were defined with a written description and presented with illustrative quotes to provide context and show their relevance.

### Researcher positioning

The researchers who conducted the interviews have a background in public health (LMM) and health psychology (REH), including previous experience of delivering behaviour change support in community settings (REH); allowing further understanding of the type of behaviour change support delivered to people who are at risk of developing long‐term health conditions. The wider team (DPF, REH, LMM) have worked on the evaluation of the face‐to‐face and digital evaluations of the NHS Diabetes Prevention Programme for the last 5 years at the time this analysis was written. DPF has worked on T2DM research projects over the last 20 years and CD has expertise in clinical and qualitative methods to develop, evaluate and implement services across a number of public health priority areas including T2DM self‐management. The backgrounds of the wider team may have influenced the interpretation of some of the findings (e.g., with more of a focus on individuals rather than wider socioeconomic constraints).

Authors DMA and CD were independent from the wider study team, thus provided new insights on the data by viewing the data independently from previous work conducted by the team. The lead author (DMA), who has a background in health psychology, therefore provided an independent perspective on service users' experiences of the programme, whilst the wider team (DPF, LMM, REH) were able to situate the findings with previous work published on the NHS‐DDPP.

### Ethical considerations

This work was reviewed and approved by the North West Greater Manchester East NHS Research Ethics Committee (Reference: 17/NW/0426, 1st August 2017). A secondary analysis of the interview data was also reviewed and approved by The University of Bath Ethics Committee (Reference: 23‐048, 18th May 2023). Full verbal consent was obtained from all participants.

## RESULTS

The sample consisted of a fairly even spread of male and females, their ages ranged from 21 to 78 years with a median of 59 years. The majority of participants (31/45, 69%) were from least‐deprived areas (from deciles 6–10 of deprivation; Ministry of Housing, Communities and Local Government, 2019). Interviews lasted for a median duration of 46 minutes. At the time of the interviews, all participants were between 2 and 4 months into the 9‐month programme. See Table 2.

**TABLE 2:** Demographic characteristics of interview participants.

	Characteristics	Values *n* (%)
Provider of programme that participants took part in	Provider 1	12 (27)
	Provider 2	10 (22)
	Provider 3	12 (27)
	Provider 4	11 (24)
Sex	Female	24 (53)
	Male	21 (47)
Ethnicity	White British	35 (78)
	Asian	5 (11)
	White Other	3 (7)
	Black	2 (4)
Index of multiple deprivation^a^	Decile 1 (most deprived)	4 (9)
	Decile 2	1 (2)
	Decile 3	2 (4)
	Decile 4	3 (7)
	Decile 5	4 (9)
	Decile 6	7 (16)
	Decile 7	3 (7)
	Decile 8	8 (18)
	Decile 9	5 (11)
	Decile 10 (least‐deprived)	8 (18)

^a^ Index of Multiple Deprivation Scores associated with the lower super output area derived from venue postcodes, ranging from the most deprived areas in England to the least‐deprived areas in England (Ministry of Housing, Communities and Local Government, 2019).

**FIGURE 1:**
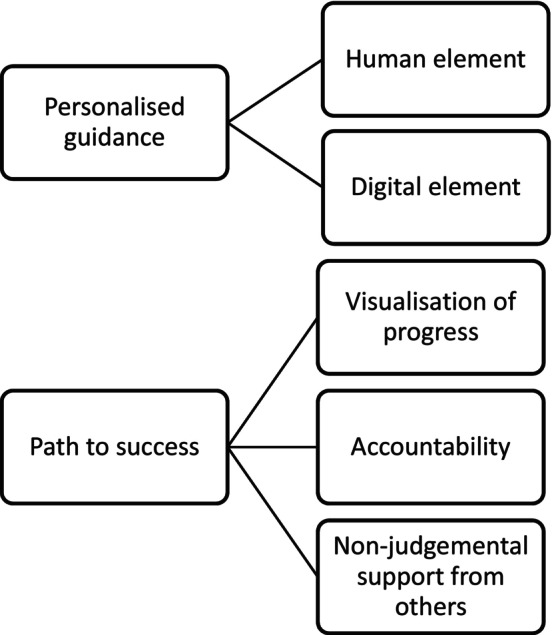
Overarching themes and sub‐themes.

Two overarching themes were developed: ‘Personalized Guidance’ and ‘Pathway to Success’, one with two sub‐themes and the other with three (see Figure 1). Quotes are presented with details of the provider programme, sex and age of each participant (e.g., Provider 2, Female, 49 years).

### Theme 1: Personalized guidance

This theme describes aspects of the programme which participants described to help them stay on track with their journeys to prevent T2DM. Participants described user‐driven personalization where they could choose the tools and components of the intervention which best suited them, akin to a ‘toolbox’. These tools guided them to making changes to their health behaviours.

### Subtheme 1.1: Human element

Participants described the health coach to provide a personal human element to the digital programme, which was particularly valued as health coaches provided participants with tailored guidance on the programme. This personalization from the health coach seemed to facilitate enablement for some participants to make changes to aspects of their health behaviours. Participants described how their health coach would provide them with specific feedback regarding their different needs based on their behaviours that they had logged in the app (e.g., diet). The health coach would suggest options and alternatives (e.g., food recipes) which guided participants towards healthier behaviours.

I think the coach thing is really invaluable, and the personalisation, which I've already said. I think that's what makes the difference. It's not just education. It's actually helping you apply it to yourself. – Provider 3, Female, 55 years

In contrast, provider 4 did not offer proactive health coach support as part of their programme. This impacted the participants' experience on provider 4's programme as they commonly expressed the importance of having this human contact despite this being a digital programme.

Yeah, so an app can only take you so far. You've got to have that human contact, probably at the start. Some people will already be there, and they just need that help, but other people who are not there kind of need that human. A phone call helps a bit, but again that physical human face, I think just can't be beaten. – Provider 4, Female, 39 years

Participants engaged with features of the programmes that best suited their needs. For example, whilst some participants did not engage with the group support feature because they either did not identify with others in their groups or they did not perceive themselves to be a ‘group person’, others found this feature helpful to keep them on track and valued hearing about other people's journeys. Interestingly, although not all users actively engaged or posted in the support groups, some described getting value from reading other people's successes which encouraged them to change their health behaviours too.

So that was a surprise, but actually I've quite enjoyed it. I've quite enjoyed seeing the various people and, you know, how well they're doing as well, I find that quite encouraging as well and a bit of a driver as well. – Provider 1, Male, 57 years

### Subtheme 1.2: Digital element

Most participants thought the educational content was well designed, informative and helpful. Despite it being described by some as ‘simplistic’, it still guided participants to make behavioural changes. Most participants reported to gain value from the food recipes, as it prompted them in the right direction of healthy eating. Many participants used the educational contents (e.g., articles, videos) which were provided digitally; this provided them with the user‐driven personalization as the materials could be accessed whenever it suited them and they could access the education topics that were most relevant. Participants described an increased awareness on how to change their behaviours after engaging with this content. Provider 3 included interactive quizzes at the end of their educational content, which participants described to stimulate learning and reinforce their knowledge.

I was impressed in the learning materials how positive and helpful the stuff's been, so actually there's been loads of stuff in terms of, you know, suggestions for recipes and things, which are very practical ways of achieving what you want, as well as background about what's going on. – Provider 3, Male, 70 years

In contrast, some participants, especially from provider 4's programme, felt that the digital content was too focused on weight loss and not focused enough on the prevention of T2DM specifically. Thus, the guidance was less helpful as it was not tailored for those participants who did not need to lose weight but were at risk of T2DM.

What I've got is a generic weight loss programme. When I didn't really need a generic weight loss programme. – Provider 4, Male, 64 years

The app was used regularly by all participants; the goal setting and tracking components of the apps were reported to be used the most. These features guided participants to change their behaviours in a personalized way, as they could choose which behaviours to log and which goals to set. These tools were described as helpful and straightforward to use by participants, especially when the app had the function to track food by taking photos, which enabled participants to obtain direct feedback from their health coach based on what they had eaten at mealtimes. Participants described the process of monitoring to increase their awareness of their everyday behaviours. The function of the food barcode scanner included in provider 4's app was particularly valued, as this increased their awareness of ingredients in some foods, and therefore guided participants towards a healthier food consumption when they were shopping in supermarkets.

Setting a goal then bar a little bit higher and achieving that and a bit higher and achieving that, and before you realise it, you get to that goal that you could have set at the outset, the 10,000 steps a day, which you would have never have got there…. – Provider 1, Male, 53 years

What the programme covers as well is that you're supposed to take photographs of your food, your three meals a day and provide any comments on that and identify what food you're eating. You're given options to identify what sort of food you are eating, whether it's soup, fruit, bread, pasta, rice, etc. So, I mean that's actually quite good and it's easy to do…. – Provider 3, Male, 66 years

### Theme 2: Path to success

This theme describes how participants successfully made changes to their health behaviours through visualization of progress, accountability, and support from others, and highlights the main drivers of behaviour change for participants on the programme.

### Subtheme 2.1: Visualization of progress

Participants particularly valued logging their diet and physical activity behaviours and weight outcomes in the app as they could track their progress through graphs which provided them with feedback. Thus, in addition to using monitoring tools to increase their overall awareness of specific health behaviours, these self‐monitoring tools also helped them visualize their progress in an accessible format. By using these graphs, participants described being able to adjust their behaviour to either get back on track or continue on the right path towards achieving their desired health outcomes.

Um, it's quite important *[the self‐monitoring feature]* because I think it, you know, it gives an indication of what you have been eating, so you can use that as a look back to see if it is the right kind of things you've been eating or doing. – Provider 3, Male, 54 years

Oh that's really important *[the self‐monitoring feature]* I think, without that you wouldn't ‐ you wouldn't keep on track, well I personally wouldn't keep on track if I didn't do that. I do have days where you eat differently because you're perhaps away from home or something's happened. But then just that little pip up *[on the graph]* makes you go back down to stick to it a bit more, doesn't it? Or if you haven't exercised so much for a reason, you can see the difference. – Provider 1, Female, 53 years

Most participants valued seeing the progress they were making in the apps, such as their weight loss and number of steps. However, many participants expressed their desire to also have regular blood glucose tests from their GP to provide them with a clearer visualization of their long‐term goal of reducing their risk of T2DM. This was particularly true for those participants who were a healthy weight, but had increased blood glucose levels. Without having any indication of their blood glucose levels, it was difficult for participants to judge whether their new habits and behaviours were working towards diminishing the risk of T2DM.

… we're talking sugar levels, the only way you can test that is a blood test, and if you only get one of those once a year you're thinking, well I think I'm doing well, I think I'm doing everything I should be doing. But I won't know for twelve months. – Provider 4, Male, 76 years

### Subtheme 2.2: Accountability

Some participants described accountability to another person to encourage them to make desired behavioural changes. For participants on provider 3's programme, they described this accountability to their health coach as they set and agreed on goals with their coach at the start of the programme. This agreement and regular contact with the health coach encouraged participants to keep on track and continue with the programme. The next time they had an encounter (via the app or telephone) with their health coach they wanted to be able to say they had successfully implemented the behaviour changes they agreed to.

Yeah, very useful *[the health coach feature]*, I think it ‐ because you have to, you know, you've got to set some goals, and you know that you know in a month's time she's going to say how did it go, you feel like you want to do it because you want to have that progression in it. – Provider 3, Female, 59 years

In contrast, participants from other provider programmes did not mention this accountability towards their health coach. Instead, some described accountability when they involved their families or friends in activities which helped their journey on the programme and facilitated behaviour changes, for example, cooking family meals, or meeting friends to exercise with.

Well it makes it an unbreakable appointment, so there's no question of looking out of the window and saying, “Oh, it's not that great a day. I won't bother.” You have an appointment, you've got to turn up. You know when you run yourself, or take some exercise like that, you might feel a twinge in your calf or have a bit of a cough and think, oh, I'll just ‐ I'd better not go any further. You don't feel like that if you're running with someone. – Provider 4, Male, 64 years

### Subtheme 2.3: Non‐judgemental support from others

Participants also described the importance of receiving support from their health coach, their family and friends. For most participants, the health coach had always been there to offer non‐judgmental advice. Participants described feeling that they could be open and honest about their attempts in making behavioural changes, even when some of these attempts were unsuccessful. Thus, in addition to providing accountability, the health coach was perceived as a sounding board and described as a credible source outside of their own social circle who participants could trust to guide them towards their desired health outcomes.

I think it's really important. I think it's nice to have somebody you trust and have somebody who's not related to you or involved with you. Yeah, to have somebody that is monitoring that with you and commenting on your progress. I think it's really useful because they're not emotionally attached to you, you feel like you can be more honest with them. – Provider 2, Female, 51 years

Just overall non‐judgemental encouragement of trying ‐ someone actually is on side trying to help you … It's more ‐ she's on side with you rather than, um, being ‐ putting judgement on you. Very encouraging, very personal, a lot of the discussion's very personal. You feel like she's trying to use any bit of knowledge she has to help you. – Provider 1, Female, 53 years

Provider 4 did not have this type of support from the health coach, but some participants on provider 4's programme described receiving support from their social circles; also reported by participants from other provider programmes. This type of support encouraged participants to maintain their behavioural changes and continued support from family and friends made following the programme feel more achievable. Participants also described the positive knock‐on effects that attending the NHS‐DDPP had on subsequent family members and friends. For example, some described that their participation in the programme led to the whole family making changes to their diet (e.g., healthier evening meals) and others described the practical support from friends who accompanied them on walks or to fitness classes.

Yeah, I get lots of support from my wife. She's keen on what stuff I eat. It's not just for me, she's keen on the whole family eating fresh stuff and healthier stuff, so it's kind of helped her as well so she's been a big support. – Provider 1, Male, 44 years

Also the friend I've got who's lost quite a lot of weight because she wanted to, we've been trying to go out at the same time, so we're chatting to each other to encourage each other to increase our activity as well. – Provider 4, Female, 67 years

## DISCUSSION

Service users' descriptions of their experiences and engagement with the NHS‐DDPP have highlighted features of the programme that helped them to change their health behaviours, and the mechanisms which facilitated those changes. Participants particularly valued receiving tailored and personalized support, both through the digital programme features (e.g., tracking tools and graphs) and contact with health coaches. Having interpersonal contact with people including a health coach, other users on the programme (via group support), or friends and family outside of the programme seemed to be vital for participants' success. When participants visualized their progress of different behaviours (e.g., food intake, steps) and outcomes (e.g., weight), it facilitated their behaviour changes. Emotional support and a sense of accountability towards their health coach or their family and friends was a driver for participants to continue with their behaviour changes.

### Contextualization of findings in the literature

Previous research on the NHS‐DDPP found that self‐monitoring was the most popular feature that users engaged with on the apps (Hawkes, Miles, Ainsworth, et al., 2023). The current qualitative research adds further context to these findings; participants reported to find self‐monitoring on the apps useful as it allowed them to visualize their progress and adjust their behaviours based on feedback of what they had logged (e.g., from a health coach or graphs generated in the apps). Alongside self‐monitoring, participants found features of setting goals and receiving feedback as key to their success on the programmes. Past research on face‐to‐face T2DM programmes found that participants were more likely to change their behaviours when they prioritized these three features (Borek et al., 2019). This suggests that digital T2DM prevention programmes can also successfully implement and deliver the same BCTs as face‐to‐face interventions (Borek et al., 2019; Van Rhoon et al., 2020).

By contrast, a qualitative study found that digital features of a T2DM self‐management programme did not seem to suit all participants with different needs and preferences (Baptista et al., 2020). This was also true for some participants on the NHS‐DDPP, for example, a minority did not engage with the educational content on the app as it was too weight loss focused and others did not engage with the self‐monitoring features due to lack of time. This can raise a concern that these digital features (like goal setting, self‐monitoring, feedback and educational content) do not work with all populations. However, current findings suggested that participants valued being able to choose which digital features worked for them; this user‐driven personalization facilitated enablement in making changes to specific health behaviours.

Support from the health coaches were particularly valued by participants in this study. Most of the provider's digital programmes included a health coach, which participants reported as a driving factor to remain engaged with the programme. Past literature on digital health interventions has also found human support to be an important component in the effectiveness of such interventions (Mohr et al., 2011; Santarossa et al., 2018). A systematic review found that interventions which had automated messages did not have a significant effect on user behaviour change, whereas interventions with text messaging and phone calls had a small‐to‐medium effect on behaviour change (Webb et al., 2010). Another systematic review of T2DM prevention programmes found that behaviour change was more successful when feedback was given by a human compared with when feedback was automated (Van Rhoon et al., 2020), suggesting that this human contact from a credible source might be crucial for fostering accountability and enhancing the overall success of digital T2DM prevention programmes.

A novel finding that this study adds to the existing literature evaluating the NHS‐DDPP is the importance that participants placed on support from their family and friends. Those in participants' social circles provided both practical support (e.g., attending fitness classes) and emotional support which participants reported to help with initiating and continuing with their behavioural changes during the first 4 months of the programme. Previous research that has interviewed service users in the pilot of the NHS‐DDP face‐to‐face programme in 2016 also found that people placed great importance on the social connections they made from meeting people on the programme and the support they had outside of the programme (e.g., by implementing changes as a family) (Rodrigues et al., 2020). Family relationships have been shown to be a big influence on the management and outcome of interventions for childhood obesity (Watson et al., 2021). This study suggests that those same social connections are also important for people taking part in digital interventions. Such findings have been echoed in a study exploring user experiences of a T2DM app for women with previous gestational diabetes (Ekezie et al., 2021), where participants reported to meet up with other app users outside of the programme and suggested that the app could be extended to close family and friends for additional support and accountability. Thus, findings from this study contribute to the literature suggesting that digital interventions could utilize human interaction to enhance people's confidence with making changes to their health behaviours and support engagement with behaviour change maintenance.

### Strengths and limitations

Participants were interviewed when they were 2–4 months into the programme, therefore, this study does not shed light on participants' experience and engagement later in the programme or whether their experiences were the same towards the end of the 9‐month programme. Further, given that the recruitment for this study took place during the COVID‐19–related restrictions, and given that the participants who had used the programme would have used it during the pandemic, these may have impacted how people engaged with the programme. We did not interview people who dropped out of the NHS‐DPP during the first 4 months; these users might have different needs that were not covered by the programme, thus the current study's findings might not be transferrable to all service users on the NHS‐DDPP.

The current study is independent research of a nationally implemented intervention, thus the research team was external to the NHS and the programme's providers. A PPIE group were involved throughout this study which enhanced the rigour of the research; we provide further reflections of PPIE across the entire research project in a separate publication (Hawkes, Sanders, Soiland‐Reyes, et al., 2023). The lead researcher in the current study was not part of the research team who conducted a wider evaluation of the NHS‐DDPP. This allowed the researcher to view the data independently and provided a new perspective by interpreting the data with no preconceptions of previous findings. A stratified purposive sampling technique was used to achieve a diverse sample size of 45 participants, which allowed for a wide spread of views from different sociodemographic backgrounds. A sufficient sample of participants from each provider programme allowed further exploration into how users' experiences varied with differences in how digital features were delivered.

### Practice implications

When the digital NHS‐DDPP is recommissioned, NHS England could emphasize in their Service Specification the importance of a proactive health coach for provider programmes. The NHS‐DDPP providers could also try to guide users to seek support from friends and family alongside the digital programme, given that this type of support was important for some participants to foster accountability and aid behavioural changes. The NHS‐DDPP might also want to consider offering a blood glucose test for users, to provide them with a clearer view on whether their behavioural changes are reducing their T2DM risk. This research has also emphasized the importance of user‐driven personalization, whereby users valued being able to use the digital features of the programme that best suited their own needs. Thus, when people are referred to the programme and given a choice between attending a face‐to‐face or digital programme, emphasizing this personalization aspect of the NHS‐DDPP may help to engage those people who experience particular barriers to attending the face‐to‐face programme such as time commitments.

These findings emphasize how participants found goal setting, self‐monitoring and feedback features particularly important; these features have been also emphasized as important by guidance documents which informed the Service Specification of the NHS‐DDPP (NICE, 2017). However, past research has been conducted on white and well‐educated individuals from higher socioeconomic status within trial settings rather than real‐world service users (French et al., 2023), therefore, the effectiveness of such features on other populations was unknown. Nevertheless, the findings from the current research suggests that participants at risk of T2DM found these features helpful, hence findings can be applicable to other digital health interventions aimed at changing health behaviours.

### Research implications

Research exploring users' experiences and engagement at the end of the 9‐month programme could provide further useful insight. A longitudinal study observing two time points could also help to understand why users reduced engagement towards the end of the programme, as found in a previous study (Hawkes, Miles, Ainsworth, et al., 2023). A recent qualitative evidence synthesis on the maintenance of behavioural changes post self‐management interventions for people living with T2DM also found that social connections (e.g., support from peers and family) and support from health care professionals are crucial for external accountability, but feeling unsupported post‐intervention contributed to behavioural relapses (Carvalho et al., 2024). Thus, longer‐term studies should continue to explore the role of social support for behaviour change maintenance.

Further, future research should explore why some service users drop out or disengage with digital interventions such as the NHS‐DDPP, which could bring new suggestions on how to make digital programmes suitable for a wider population. This could be achieved by conducting focus groups with specific user groups who may be less engaged with digital interventions (e.g., older people, people from ethnic minority backgrounds) to better understand their barriers to engagement and how these interventions can be better tailored to the needs of these groups. Future research could also explore the actual usage of the NHS‐DDPP by participants before interviewing them. This could provide some insight into useful intervention components in the programme, and aspects of the programme which are not recalled could highlight those components which may not aid behavioural changes.

## CONCLUSION

This study has highlighted important features which could be beneficial to implement in digital health behaviour change interventions targeting T2DM prevention. Digital interventions that aim to encourage changes in health behaviours could consider how to incorporate support from health coaches, friends and family to foster accountability and behaviour change maintenance. Additionally, interventions that can be customized to best suit individual users' needs could potentially improve programme success.

## AUTHOR CONTRIBUTIONS

**Davide Moussas‐Alvarez:** Writing – original draft; formal analysis. **Rhiannon E. Hawkes:** Methodology; validation; investigation; data curation; supervision; writing – review and editing; project administration. **Lisa M. Miles:** Investigation; methodology; writing – review and editing; project administration; data curation. **Charlotte Dack:** Supervision; writing – review and editing. **David P. French:** Funding acquisition; conceptualization; supervision; writing – review and editing.

## FUNDING INFORMATION

This research is funded by the National Institute for Health and Care Research (The Health and Social Care Delivery Research (HSDR) Programme, 16/48/07 – The Evaluating the NHS Diabetes Prevention Programme [NHS‐DPP]: the DIPLOMA research programme [Diabetes Prevention: Long‐Term Multimethod Assessment]). The views and opinions discussed in the present study are those of the authors, and do not necessarily reflect those of the National Institute for Health and Care Research or the Department of Health and Social Care.

## CONFLICT OF INTEREST STATEMENT

The authors declare that they have no competing interests.

## Supporting information


File S1.

File S2.

File S3.

File S4.

File S5.

File S6.

## Data Availability

Transcripts of interviews analysed in the current study are not publicly available due to confidentiality agreements with the provider organizations, as some information is commercially sensitive. Some datasets are available from the corresponding author on reasonable request, although authors will require the explicit permission of the relevant provider organizations.
