# Supplementary material for: Service users' experiences of, and engagement with, a nationally implemented digital diabetes prevention programme
Source: Br J Health Psychol. 2025 Feb 19;30(1):e12787. doi: 10.1111/bjhp.12787 (PMC11837234; doi:10.1111/bjhp.12787)
Supplement: Supplementary file 2 — File S2. [file BJHP-30-0-s006.docx]

**Supplementary File 2: Participant recruitment**

|  | **Provider 1** | **Provider 2** | **Provider 3** | **Provider 4** |
| --- | --- | --- | --- | --- |
| **Number invitations sent in first round** | 59 | 112 | 33 | 184 |
| **Number of invitations sent in second (and third) round** | 130 | 7 (+17) | 0 | 38 |
| **Total number of invitations sent** | 189 | 136 | 33 | 222 |
| **Number of participants who got in contact to take part** | 21 | 14 | 13 | 20 |
| **Number of participants who did not proceed for interview, including reasons** | 9  N=9 no contact | 4  N=4 no contact | 1  N=1 not eligible | 9  N=6 no contact  N=2 not eligible  N=1 declined |
| **Final number of participants** | 12 | 10 | 12 | 11 |
