# Supplementary material for: Service users' experiences of, and engagement with, a nationally implemented digital diabetes prevention programme
Source: Br J Health Psychol. 2025 Feb 19;30(1):e12787. doi: 10.1111/bjhp.12787 (PMC11837234; doi:10.1111/bjhp.12787)
Supplement: Supplementary file 3 — File S3. [file BJHP-30-0-s003.docx]

**Supplementary File 3:
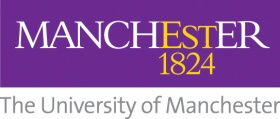
Interview Schedule**

**Evaluating the Digital NHS Diabetes Prevention Programme**

**Question schedule 1**

Thank you for agreeing to take part in this interview. This is the first interview of two that we hope to have with you. Please let me know if you would like to take a break or pause the recording at any point, or if you would like to stop the interview. Please note the information we collect will be kept securely and confidentially in accordance with data protection law, as described on the participant information sheet. The interview should take no more than one hour.

**Questions about the Digital ‘Healthier You’ Diabetes Prevention Programme**

1. How were you directed towards taking part in the digital Diabetes Prevention Programme?
2. Before you started the ‘Healthier You’ digital sessions, what did you expect from the online course?
   1. Was the online course similar or different to what you were expecting?
3. What do you think the main aim of the course is?
   1. How does it try to do that?
   2. Was this made clear to you at the start of the course?
4. How often have you been accessing this online programme so far?
   1. Is this what you expected?
   2. What would encourage you to engage with and access the programme more often?
5. Can you describe the support you received when you were first enrolled onto the digital programme?
   1. What were you asked to do during your first phone call with the health coach?
      1. Introduction to digital programme?
      2. Goal setting?
      3. Tracking behaviours/weight?
   2. How did you feel after this initial phone call with your health coach?
   3. What contact have you had with the health coach since this initial phone call?
6. What information have you been given on the course so far about diabetes and preventing diabetes?
   1. Was it the right type, amount, format, level of difficulty?
   2. Is there any information that has encouraged you to change aspects of your current lifestyle?
7. What activities have you been asked to compete so far?
   1. Reading?
   2. Interactive content (e.g. videos, worksheets, external websites)?
   3. Interaction with health coach?
   4. Interaction with others also taking the course?

**(Elicit description of components, then in turn):**

- - **Have you completed this?**
  - **What do you think this activity was trying to achieve?**
  - **How useful did you find this for helping to change your diet and physical activity?**

1. What support have you received on the programme so far?
   1. One-to-one coaching?
   2. Group support from others on the programme?
   3. Support from family and friends?
   4. External websites?

**(Elicit description of components, then in turn):**

- - **How have you found this support?**
  - **How useful did you find this support for helping to change your diet and physical activity?**

1. So far, have you been asked to set any goals or targets for your physical activity, or diet, or weight loss?
   1. How are you getting on with that? (Choosing, setting, working towards goal?)
   2. How do you think goals might *work* to help people change their physical activity or diet?
   3. How *useful* are you finding this for changing your physical activity or diet?
2. Have you been asked to make a more detailed plan for changing your diet or physical activity, sometimes called an action plan?
   1. How are you getting on with that? (Making a plan, sticking to it?)
   2. How do you think action plans might *work* to help people change their physical activity or diet?
   3. How *useful* are you finding this for changing your physical activity or diet?
3. Have you been asked to keep track of your physical activity or diet or weight, by using a daily diary/log or a step counter?
   1. How are you getting on with that?
   2. How do you think keeping track of things in this way might *work* to help people change their physical activity or diet?
   3. How *useful* are you finding this for changing your physical activity or diet?
4. Have you been asked to think about any barriers that get in the way of making healthy changes and come up with solutions to getting over these barriers?
   1. How did you get on with that?
   2. How do you think problem solving in this way might *work* to help people change their physical activity or diet?
   3. How *useful* did you find this for changing your physical activity or diet?
5. Have you been given any feedback so far on changes to your weight, blood glucose levels, diet or physical activity?
   1. How *useful* did you find it to have that feedback?
   2. How do you think getting feedback in this way might *work* to help people change their physical activity or diet?
   3. How did you feel when you were given that feedback?
   4. How timely was the feedback?
6. Has there been anything else on the course so far that you have found useful?
7. Is there anything else about the course we haven’t talked about yet that you’d like to tell me about or report back on?

*Thank you very much for taking the time to take part in our research.*
