# Supplementary material for: Service users' experiences of, and engagement with, a nationally implemented digital diabetes prevention programme
Source: Br J Health Psychol. 2025 Feb 19;30(1):e12787. doi: 10.1111/bjhp.12787 (PMC11837234; doi:10.1111/bjhp.12787)
Supplement: Supplementary file 4 — File S4. [file BJHP-30-0-s001.docx]

**Supplementary File 4: Patient and Public Involvement in this study, and the wider programme evaluation**

*Reported using the GRIPP2 short form reporting checklist (Staniszewska et al., 2017).*

*For more detailed reflections on patient and public involvement across the entire programme of research, see Hawkes et al. (2023).*

| **Section and topic** | **Description** |
| --- | --- |
| **1. Aim**  Report the aim of PPIE in the study | The aim of the PPIE group for this specific interview study was to provide feedback on the questions drafted for the topic guide. The group also aimed to provide input throughout the study duration, e.g., through providing feedback on provisional qualitative findings. |
| **2. Methods**  Provide a clear description of the methods used for PPIE in the study | Members of the PPIE group (n=4 people at risk of or living with type 2 diabetes; n=2 female and n=2 male), met with the research team via videoconferencing for a 1.5 hour meeting. The PPIE group provided feedback on the wording and order of the interview questions.  In subsequent meetings, after the interview data had been collected and the researchers had conducted preliminary analyses (exploring how users understood and used the behaviour change content; the primary analysis), the research team presented these findings to the PPIE group. The group commented on further findings describing user experience and engagement that would be of most interest to members of the public and other key stakeholders; it was these insights that we report for this secondary analysis. |
| **3. Study results**  Outcomes – report the results of PPIE in the study, including both positive and negative outcomes | The PPIE group advised on the ordering of the questions, for example, they advised to ask the more ‘cognitively demanding’ questions later on in the interview (including the questions about participants’ understanding of specific behaviour change techniques).  The group advised on the wording of some of the questions and any additional questions to ask. For example, the following question was added to the topic guide after meeting with the PPIE group: “Before you started the ‘Healthier You’ digital sessions, what did you expect from the online course?”  The group further suggested that interviewees may need breaks factored into the interview. We therefore added the following prompt for researchers to mention at the start of the interview: “Please let me know if you would like to take a break or pause the recording at any point, or if you would like to stop the interview.”  Following the second meeting with the PPIE group, and based on the group’s insights into further findings describing user experience and engagement that would be of interest to other key stakeholders, the research team conducted a secondary analysis of the interview data with these research questions in mind. These insights are reported in the current analysis.  We have further worked with the PPIE group to produce lay summaries of research findings, including animation videos with findings across the entire research project. These have been disseminated via various channels and can be found on the project website: <https://arc-gm.nihr.ac.uk/projects/diploma-evaluation-national-nhs-diabetes-prevention-programme> |
| **4. Discussion and conclusions**  Outcomes – comment on the extent to which PPIE influenced the study overall. Describe positive and negative effects | Involving public contributors early during the research study ensured that this study was acceptable to potential participants, and is considered a best practice approach.  The regular dialogue between the research team and PPIE group ensured that public contributors were able to input on study results and suggest ways to present the results to a wider audience. |
| **5. Reflections/ critical perspective**  Comment critically on the study, reflecting on the things that went well and those that did not, so others can learn from this experience | PPIE has been vital throughout the life of the wider research project, from study design through to dissemination of findings. Members of the group provided a unique and broader perspective on the work, which increased the potential of this research to meet the needs of patients and members of the public.  However, PPIE contribution for a commissioned research evaluation of an NHS service already implemented had unique challenges. For example, it was not within the remit of the independent research team to make changes to the NHS Diabetes Prevention Programme itself. This approach was much less direct than what some contributors might have been used to.  Given the reduced scope for our PPIE group to provide direct feedback on the programme itself, we were keen to give at least equal focus to ‘engagement’ as well as ‘involvement’, to produce accessible materials on findings for the public. We view this as a particular strength of our approach as this gave the PPIE a higher profile than might be the case with other research projects. |

**References:**

Hawkes, R. E., Sanders, C., Soiland-Reyes, C., Brunton, L., Howells, K., Cotterill, S., ... & Bower, P. (2023). Reflections of patient and public involvement from a commissioned research project evaluating a nationally implemented NHS programme focused on diabetes prevention. *Research Involvement and Engagement*, *9*(1), 42.

Staniszewska, S., Brett, J., Simera, I., Seers, K., Mockford, C., Goodlad, S., ... & Tysall, C. (2017). GRIPP2 reporting checklists: tools to improve reporting of patient and public involvement in research. *BMJ*, 358.
