# Supplementary material for: Service users' experiences of, and engagement with, a nationally implemented digital diabetes prevention programme
Source: Br J Health Psychol. 2025 Feb 19;30(1):e12787. doi: 10.1111/bjhp.12787 (PMC11837234; doi:10.1111/bjhp.12787)
Supplement: Supplementary file 5 — File S5. [file BJHP-30-0-s002.docx]

**Supplementary File 5: Coding Framework**

1. **How do service users describe their engagement with the NHS-DDPP?**

| Generic categories | Codes | Code Description |
| --- | --- | --- |
| Engagement with health coach | **Engages with HC nearly every day** | *The extent to which participants engage with their health coach (codes part of the text when they talk about their HC engagement).* |
|  | **Engages with HC weekly or monthly** |  |
|  | **Does not engage with HC** |  |
| Engagement with group support | **Writes messages on chats or forums** | *The extent to which participants engage with their group support that is delivered through chats and forums.* |
|  | **Observes chats or forums** |  |
|  | **Does not engage with chats or forums** |  |
| External support | **Family and friends support** | *When they get some sort of support from their partner friends or family, which can either be their family joining them in their activities or just being supporting about them tacking the programme.* |
| Educational content | **Engages with articles or videos** | *Participants engagement with the educational content the programme provides.* |
|  | **Does not read or watch educational content** |  |
| App | **Engages with logging features of the app** | *The extent to which participants engage with features of the app, such as logging their steps, weight, food consumption etc.* |
|  | **Does not engage with some features of the app** |  |
| Technology | **Uses starter package** | *Participants are given a welcome package, so code relatively to if they use or do not use this.* |
|  | **Does not use any of the gadgets given** |  |
| Overall programme engagement | **Immediate contact by provider** | *The extent to which participants are contacted and also if they engaged with the overall programme.* |
|  | **Delayed contact by provider** |  |
|  | **Not engaging with the programme** |  |

1. **What are the service users’ experiences with the NHS-DDPP?**

| Generic category | Codes | Code Description |
| --- | --- | --- |
| Positive experience with health coach | **Good relationship with HC** | *Participants feels supported by health coach, they are offered plenty of emotional support and gives helpful feedback.* |
|  | **Personalisation of the programme guided by HC** | *Participants feels that it is easy to communicate to the HC, HC guided them to set goals which are not strict, and they feel that the HC is tailoring to programme to the participant.* |
| Negative experience with health coach | **Negative communication with HC** | *Participants either have not got any contact with their health coach or they do not have to amount of support or responsiveness from them.* |
| Group support | **Valuable group support** | *Participants felt that they were helped by the support given through group support and they could relate to the experiences of other users in these groups.* |
|  | **No happy about their group support** | *Participants were unable to relate to the group they were assigned for their group support.* |
| App | **Enjoyed using the app** | *Participants enjoyed using features of the app like setting goals, ‘habits’, adding their weight, adding pictures of their foods etc. Also enjoyed the variety of features the app provided.* |
|  | **Not happy about the app** | *Participants were not happy about the content provided to them, as it might have been too generic or not as tailored and the app interface was too complex.* |
| Technology | **Enjoyed receiving and using the welcome package** | *The extent to which participants found the welcome pack useful or not for their journey in this programme.* |
|  | **Found the welcome package complicated or useless** |  |
| Educational content | **Positive experience with educational content** | *Participants had a good experience with the educational content of the programme, such as: they found it useful as it facilitated their behaviour change, it was easy to access and they had a flexibility on what to access, happy about the content not being directly related to diabetes, enjoyed reading articles, etc.* |
|  | **Negative experience with educational content** | *Participants had a bad experience with the educational content of the programme, such as: they found it way too long and too basic, they were overwhelmed by the content which was sent to them, etc.* |
| General experiences | **Barriers and initial challenges** | *Participant that faced a barrier during or at the start of their interventions with various aspects of the programme (e.g. logging steps, food, weight etc).* |
|  | **Tailored experience** | *Participants that felt that either the health coach or the programme allowed for flexibility and allowed for them to tackle their specific needs.* |
|  | **Good overall experience** |  |
|  | **Bad overall experience** |  |
| Comments | **Suggestions** | *Participants when they pointed out how any aspect of the programme could have been different or changed.* |
